# Can Music Foster Learning – Effects of Different Text Modalities on Learning and Information Retrieval

**DOI:** 10.3389/fpsyg.2017.02305

**Published:** 2018-01-09

**Authors:** Janina A. M. Lehmann, Tina Seufert

**Affiliations:** Department of Learning and Instruction, Ulm University, Ulm, Germany

**Keywords:** learning with music, melody as a mnemonic, reading comprehension, listening comprehension, background music, working memory

## Abstract

This study investigates the possibilities of fostering learning based on differences in recall and comprehension after learning with texts which were presented in one of three modalities: either in a spoken, written, or sung version. All three texts differ regarding their processing, especially when considering working memory. Overall, we assume the best recall performance after learning with the written text and the best comprehension performance after learning with the sung text, respectively, compared to both other text modalities. We also analyzed whether the melody of the sung material functions as a mnemonic aid for the learners in the sung text condition. If melody and text of the sung version are closely linked, presentation of the melody during the post-test phase could foster text retrieval. 108 students either learned from a sung text performed by a professional singer, a printed text, or the same text read out loud. Half of the participants worked on the post-test while listening to the melody used for the musical learning material and the other half did not listen to a melody. The written learning modality led to significantly better recall than with the spoken (*d* = 0.97) or sung text (*d* = 0.78). However, comprehension after learning with the sung modality was significantly superior compared to when learning with the written learning modality (*d* = 0.40). Reading leads to more focus on details, which is required to answer recall questions, while listening fosters a general understanding of the text, leading to higher levels of comprehension. Listening to the melody during the post-test phase negatively affected comprehension, irrespective of the modality during the learning phase. This can be explained by the seductive detail effect, as listening to the melody during the post-test phase may distract learners from their main task. In closing, theoretical and practical implications are discussed.

## Introduction

Research in the field of learning and instruction aims to investigate how learning processes can be fostered. Comparisons between different text modalities are a broadly investigated topic (e.g., Penney, 1989; Pächter, 1997; Rubin et al., 2000). During the last years, learning with music also received a lot of attention (e.g., Lehmann and Seufert, 2017; Schwartz et al., 2017). Research in this area mostly focussed on background music, i.e., learning a text while listening to music (for a meta-analysis, see Kämpfe et al., 2010). However, music, more specifically the lyrics, can also be considered as a medium to convey academically relevant information. Learning text in a sung modality also provides the possibility to use the melody as a mnemonic aid to ease text retrieval. Whether music can really foster learning compared to learning with spoken and written text is examined in this paper.

### Processing Auditory and Visual Information

Investigations into the differences in learning outcomes between written, spoken, and sung texts is based on the notion that all three modalities are processed differently, especially in working memory and therefore foster different levels of text processing (Baddeley, 1986; Mayer, 2001).

The cognitive theory of multimedia learning by Mayer (2001) explains that auditorily and visually presented learning materials (from now on referred to as auditory and visual learning material) differ regarding their modality. Besides using the ears for listening to spoken text and the eyes for reading written texts, Mayer (2001) also assumes that the processing of both presentation modes differs: in general, auditory text is processed by the auditory channel in working memory, while visual information is processed by the visual channel (dual-channel assumption). However, an experienced reader is able to mentally convert the visual surface of the text into sounds. These sounds are then processed by the auditory channel. The dual-channel assumption is based on the model of working memory by Baddeley (1986).

Baddeley (1986) assumes the presence of two different subsystems, which are controlled by the central executive: the visuospatial sketchpad, and the phonological loop. Words are processed by the two subsystems. The pictorial surface of printed letters needs to be encoded as spatial information within the visuospatial sketchpad. The information is then transferred to the phonological loop, where it takes on a semantic meaning. This translation process is not necessary for auditory information. The auditory information can be processed directly within the phonological loop, which requires less cognitive capacity (van Dijk and Kintsch, 1983).

However, the processing of visual and auditory texts does not only differ with regard to the memory structures involved, but also with regard to the workload that the different modalities produce. This is even more important as working memory capacity is limited to four units for processing or learning (Cowan, 2001). As mentioned above, reading needs one additional processing step compared to learning from auditory information (van Dijk and Kintsch, 1983). Moreover, reading also requires cognitive capacity for the control of eye movements (Kürschner et al., 2006; Rummer et al., 2008). Nevertheless, visual information is stable compared to transient auditory information so that with printed texts there are more possibilities for learners to control and self-regulate the learning process (Pächter, 1997; Kürschner et al., 2006). Learners can individually determine their reading pace, and repeat sentences or even whole paragraphs if they need to. This leads to the question of whether this advantage of self-regulation possibilities really pays off and can be shown empirically.

Empirical evidence from comparisons of auditory and visual learning material, shows the importance of considering text complexity: Spoken words are superior to visual words in recall and comprehension only when learning short texts with low complexity (Penney, 1989; Pächter, 1997; Rubin et al., 2000). This is due to the auditory recency effect (Penney, 1989; Rummer et al., 2008). Auditory words lead to an acoustic-sensory representation, which can be compared to a mental echo and relates to perceptual processing, not to working memory processes. Furthermore, listening to a short text leads learners to focus on the gist of the text and subsequently to better overall understanding and coherence formation (Rubin et al., 2000). In contrast, when learning more complex texts, visual presentations lead to better learning outcomes (Müssler et al., 1985), because self-control possibilities become increasingly important with increasing text complexity. This also leads to a stronger focus on the details and the propositional inferences of a text (Rubin et al., 2000). Based on these results, one might conclude that to promote learning, easier text should be presented auditorily while more complex texts should be presented in written form.

In fact, we propose that the relationship described above – listening leads to better understanding, reading to more focus on the details – can be transferred to the different levels of learning outcomes. Such levels have been classified, for example, by Bloom (1956) or van Dijk and Kintsch (1983). As focussing on memorizing details should foster the ability to reproduce text, learning with written text should be especially beneficial for answering recall questions. On the other hand, focussing on gist and coherence formation should foster deeper processing and elaboration and therefore, auditory texts should foster a learner’s performance when answering comprehension questions (Rubin et al., 2000).

### Processing Musical Information

Sung text is also auditorily presented, but its processing differs from that of spoken words. The theoretical model of how music is processed in working memory (Berz, 1995) assumes that an independent subsystem exists. Berz’s (1995) model is based on Baddeley’s (1986) model of working memory, but includes an additional specific subsystem that is responsible for the processing of music. Therefore, the phonological loop does not become overburdened by the processing of sung text consisting of auditive words and the accompanying melody. Different empirical studies validate this claim. For example, Rowe et al. (1974) tested whether the recall of words and sounds differs depending on the kind of shadowing experienced after learning. In a shadowing phase, participants were distracted from the content they had learned in an earlier learning phase. The distraction was listening to words or sounds other than those to be learned. Words were recalled better after being distracted by music rather than by poetry. In contrast, sounds were recalled better after being distracted by poetry rather than by music. Therefore, Rowe et al. (1974) theorized that verbal and musical information must be processed in different subsystems (Deutsch, 1970; Paivio et al., 1975; Salame and Baddeley, 1989; Rowe, 2013). The asymmetrical integration effect by Jamieson and Cuddy (2001) also supports the idea, that lyrics and melody are stored differently in memory. Their findings indicate that a studied melody was recognized better when presented with the matching lyrics. However, the studied lyrics were not recalled better when presented with the matching melody. Comparable results were found in a study by Peynircioğlu et al. (2008). This finding is also supported by a single-case study by Steinke et al. (2001) which reports on an amateur musician who became a music after a stroke in the right-hemisphere of his brain. His verbal skills were not affected by the stroke, which also supports the notion of different stores in memory. For people with advanced musical training, these different subsystems can also be found neurologically. Schulze and Kölsch (2012) found that different, but partly overlapping brain areas are activated while processing verbal and musical information.

If sung text is processed by two different subsystems, it is dually coded by the verbal and the melodic information. Based on the findings of the dual coding theory (Paivio, 1986; Sadoski and Paivio, 2004), one could infer that sung texts for learning should be recalled and comprehended better than single coded spoken texts. The original dual coding theory (Paivio, 1986) assumes that concrete words such as “tree” are processed with both the verbal channel and the imagery channel, and therefore simultaneously activate both working memory systems. Thus, concrete words are recalled better than abstract words such as “freedom,” which only activate the verbal system. Further studies confirmed this finding, and that comprehension outcomes also benefit from dually coded words (e.g., Sadoski and Paivio, 2004). Transferring this dual coding assumption to the processing of sung text, this would speak for an advantage of sung text over spoken text.

Furthermore, aside from the dual coding, sung text provides further advantages for learning. Each melody provides a rhythm, which can underline the importance of specific information within the lyrics (Palmer and Kelly, 1992). Moreover, rhythm eases the recall of lyrics (Hyman and Rubin, 1990). This rhythm allows the learner to build chunks by grouping information into larger musical patterns which facilitates recall (McElhinney and Annett, 1996; Thaut et al., 2005). Moreover, lyrics usually rhyme. This specific rhyming text format may lead to an even stronger feeling of rhythm. It is certainly not possible to speak without any rhythm. A spoken text has a natural rhythm due to its speech melody (Nooteboom, 1997) and the internal representation of a visual text, may tend to have a similar rhythm as well. This kind of speech melody is less complex, mostly pitched within the range of one fifth, and is much more familiar than a song’s melody as we hear and speak words every day (Fuchs and Röber-Siekmeyer, 2002). Speech can be made more rhythmical, for example by employing specific text formats, such as the rhymed verses of a rap song (e.g., Hirjee and Brown, 2009).

Besides these advantages for learning, a sung text also provides more information which has to be processed compared to the processing of spoken words, i.e., the additional information of the comparably complex melody. Processing this melody poses additional load in working memory. In a learning context, cognitive load can be caused by three different types of load: intrinsic, germane, and extraneous cognitive load (Sweller et al., 1998, 2011; Plass et al., 2010). Intrinsic load is caused by element interactivity, more specifically, the complexity of the task. Intrinsic load can be decreased by chunking processes. Germane load is due to the learner’s engagement in the learning process, whilst extraneous load refers to the design of the learning task. While processing the lyrics of a song, intrinsic load may be decreased by eased chunking due to the melody (McElhinney and Annett, 1996; Thaut et al., 2005). However, the additional melodic information also needs to be processed, raising extraneous cognitive load. In general, higher extraneous load hinders learning (Sweller et al., 1998, 2011; Plass et al., 2010). Although, singing usually takes more time than speaking a text (Kilgour et al., 2000). Thus, learners have more time to process all the information. This longer presentation time combined with a decreased intrinsic load might offset the additional information of the melody, which needs to be verified empirically.

On an empirical base, a wide range of studies (e.g., Wallace and Rubin, 1991; Wallace, 1994; Purnell-Webb and Speelman, 2008; Governor et al., 2013; Ludke et al., 2014; Good et al., 2015) have found a clear superiority of sung texts over spoken texts in a variety of contexts, such as foreign language learning, learning of word lists, learning of whole texts and academic learning. Conversely, there are studies, which found completely contradictory results (e.g., Wallace, 1994; Racette and Peretz, 2007; Tamminen et al., 2016): spoken text was learned better than sung text. Due to the nature of these different learning tasks, dependent measures varied a lot. Hitherto, there has been no systematic review which considers all potential influencing variables that could explain these inconsistencies. This is due to the fact that most of the previous studies do not sufficiently describe their musical stimuli, i.e., their independent variables. Moreover, potential control variables such as the format of text (rhymes versus no rhymes), characteristics of the melody (e.g., tempo, key, or induced mood), familiarity of the melody and musical training of the participants need to be considered.

Previous studies in this field of research either collected data from university students or adults. However, high school students might also profit from the potential beneficial effect of a sung text. We expect the same cognitive mechanism to be effective for teenagers and adults alike. Nevertheless, to provide further evidence for this claim, differences between various modalities should also be investigated in this age group.

### Melody as a Mnemonic

One important question arising from the idea of dual coding is how the link between lyrics and melody could benefit learners. Both information paths are addressed simultaneously. Therefore, one can assume these are two closely linked paths in memory, when melody and text are learned in combination and when the melody is easy enough to be learned and stored in long-term memory within the given time. Another possibility would be the use of a familiar and established melody, which is already stored in long-term memory. In these cases, during information retrieval the melody could work as a mnemonic anchor, which in general is a well stored and easy-to-retrieve information path in the long term memory. Activating the anchor by presenting the melody should facilitate the recall performance of the lyrics. The phenomenon of using melodies as a mnemonic has already been used successfully in relation to jingles for advertising (e.g., Scott, 1990; Yach, 1991) or in clinical contexts, for example, while working with patients with multiple sclerosis, aphasia, or Alzheimer’s disease (e.g., Goldfarb and Bader, 1979; Moore et al., 2008; Simmons-Stern et al., 2010). Patients with such diseases can have problems memorizing information or learning new information. In such cases, music can work as a mnemonic to help when learning new information, while music also facilitates verbal learning for patients in the early stages of multiple sclerosis. Furthermore, if the learner remembers the lyrics during the test phase, this would allow him or her to think about the words more deeply and aid comprehension. Therefore, by hearing the melody during retrieval, comprehension outcomes should be fostered as well.

To test these assumptions, one has to ensure that if better learning outcomes result after learning with the sung text, this is not due to other effects of listening to music, such as motivational effects (e.g., Rey, 2012). For this reason in our experiment half of every text modality group (even those who had learned with visual or spoken words and not the sung text) needed to listen to the melody during the test phase. These learners who were unfamiliar with the melody simply listened to background music without any potential mnemonic function. In fact, background music might even distract these learners. Moreover, this kind of music is not related to the main task and is therefore, an unnecessary cognitive burden for the learner. This is known as the seductive detail effect (Garner et al., 1992; for a meta-analysis, see Rey, 2012). Seductive details often lead to split attention and cognitive overload and therefore, to worse learning performance (Chandler and Sweller, 1991; Sweller et al., 1998, 2011; Plass et al., 2010).

Research relating to the influence of background music is in keeping with the seductive details assumption: A meta-analysis by Kämpfe et al. (2010) found that background music impedes learning. However, there are also studies that found background music to have either a positive influence (e.g., Hallam et al., 2002) or no influence (Martin et al., 1988). This can be explained by the arousal-mood hypothesis (Thompson et al., 2001; Husain et al., 2002). This approach states that music has an effect on learning through a mediation over arousal and mood. Depending on the amount of arousal and on the valence of mood that is induced by the music, listening to music while learning can be beneficial or impeding. Another theoretical approach by Gendolla et al. (2001) also points out the importance of mood on achievement tasks: In their study, participants scored higher demand appraisals when being in a negative compared to a positive mood.

### Research Questions and Hypotheses

The first research question of this study is: (Q1) how do different text modalities (i.e., visual, spoken, and sung texts) influence recall and comprehension of the presented text?

– We hypothesize that there will be a main effect of *text modality* on both recall and comprehension performance (H1).
  ◦ For recall performance, we expect visual texts to be superior to spoken and sung texts; and sung texts to be superior to spoken texts (H1a).
  ◦ For comprehension performance, we expect sung texts to be superior to visual and spoken texts (H1b).

The second research question is, (Q2) whether presenting the melody of the song during the test phase affects recall and comprehension of the text presented in the learning phase.

– We expect to find no main effect (H2) of the *presentation of the melody during the test phase* for neither recall (H2a) nor comprehension (H2b).

We are especially interested in (Q3) analyzing whether learners are affected differently by the music during the test phase depending on whether they previously learned the text in combination with the music or not (sung version versus auditory or visual version).

– We assume that there will be an interaction between the two factors of *text modality* and *presentation of the melody during the test phase* (H3).
  ◦ The interaction should be visible by an increased test performance when listening to the melody during the test after learning with the sung learning material (H3.1) for recall (H3.1a) and comprehension (H3.1b).
  ◦ Hearing the melody during the test phase for both the written as well as the spoken text groups should decrease test performance (H3.2) for both recall (H3.2a) and comprehension (H3.2b).

## Materials and Methods

### Participants

We tested 112 students (61% females) from a German high school who were aged between 12 and 19 years (*M*_age_ = 16.21; *SD*_age_ = 1.34). All participants whose results in the post-test were more than two standard deviations apart from the mean were omitted and could therefore be defined as statistical outliers (e.g., Barnett and Lewis, 1994). Finally, 108 participants were included in further analysis.

### Design

We applied a 3 × 2 between-subject design. Participants were randomly assigned to a visual (*n* = 36), spoken (*n* = 38), or sung (*n* = 38) text condition (*factor 1: text modality*). Half of each group listened to the melody of the song while answering the questions about the text, while the other half did not listen to the melody (*factor 2: presentation of the melody during the test phase*).

### Materials and Measures

**FIGURE 1:**
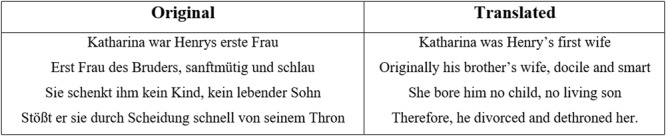
Example verse from the learning material: rhymed German version and a translated version.

The text-based *learning material* was 200 words long and comprised of six rhymed verses and a refrain related to Henry VIII (see **Figure 1**). This unusual text format was chosen because of the nature of the lyrics of songs, which usually consist of rhymed verses and refrains. To ensure comparability between all three text modalities, we used the same format (verses and refrain) for the written and spoken conditions. Due to the rhymes, both the visual and the auditory text are more rhythmical than texts in standard prose. Nevertheless, we decided to keep the same text format in each condition to avoid any confounding effects of the text format.

**FIGURE 2:**
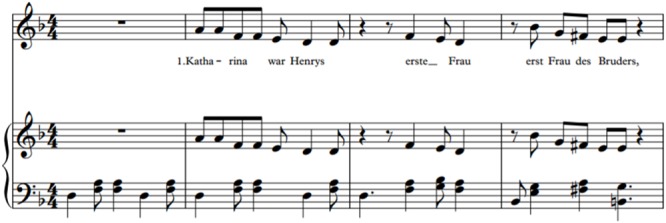
First line of the song (voice and piano score).

Participants in the visual text condition received printed learning material. Learners in both auditory conditions heard the text to be learned through headphones connected to an IPod. All other materials were provided in a printed version. The two auditive records (spoken and sung) were performed by a female voice teacher who also composed the melody of the sung version of the learning material. The melody was aimed to be simple enough to function as a mnemonic aid. The sung text had a pitch range of one octave and one semitone and was sung in soprano in a minor key. The melody of the sung version was accompanied by a monophonic piano (please see **Figure 2** for the first line of the voice and piano score). The spoken version had a pitch range of one octave and was performed by the same voice. Both records were reviewed by an independent observer for intelligibility: the observer was able to understand each word correctly. To avoid any motivational interference between groups with and without headphones and music players, each student, regardless of the condition, received their own pair. In the visual group, the spoken track only consisted of instructions to start reading the text and also informed them when to stop. The allocated reading time was the same length as the duration of the sung text (6 min 32 s). The spoken version was slightly shorter (4 min 36 s). To avoid floor effects and to provide the possibility to really learn the melody of the song and store it in long-term memory, the text was played twice in both auditory conditions.

We developed the pre-test for *prior knowledge* which comprised of five open questions (e.g., “Who was Henry VIII?”). We also developed the test for *learning outcomes* which consisted of 19 questions. 14 questions were recall tasks (e.g., “What was the name of Henry’s first wife?”) the remaining five were comprehension tasks (e.g., “What was Henry’s problem with women in general?”). Each answer was marked by comparing it to a predefined solution.

In a short *demographic questionnaire*, participants were asked for their age, gender, school grade, their mother tongue, and whether they had a diagnosed reading disability. Previous studies have found that the effects of instructional designs often depend on learner characteristics like age, sex, motivation or prior knowledge (e.g., Kalyuga et al., 2003; Seufert, 2003; Moreno, 2005). Motivation was measured with the questionnaire for current motivation (Vollmeyer and Rheinberg, 2006) consisting of the four subscales of interest, challenge, probability of success, and fear of failure. Due to the special aspect of using music in our study, we also considered learners’ experience with singing in a choir, classical music (because we chose a classical melody for our song) and with playing an instrument (Wallace, 1994) as potential influencing and confounding variables. These constructs should represent the musical training of our participants which may facilitate processing of musical information, as suggested in previous studies (e.g., Wallace, 1994; Kilgour et al., 2000; Ginsborg and Sloboda, 2007; Racette and Peretz, 2007). Both variables were measured dichotomously (e.g., “Do you sometimes listen to classical music? Yes/No”). At the end of the study, those students who listened to music during the test phase answered three questions about their *perceptions of the music*. They were asked whether they considered the music to be distracting, stimulating, or pleasant. The students ranked each feature on a Likert scale ranging from 1 (not at all) to 5 (completely).

All research related materials can be received by contacting the corresponding author.

### Procedure

Before the data collection took place, all parents of students who were under the age of 18, received a letter about the study including information about the duration and tasks of the experiment and permission was sought for their child to take part in the study. All parents consented to their child participating in the study. Participants over the age of 18 signed the informed consent themselves. The study lasted about 45 min. Participants were tested with their class during a teaching period in their classrooms. All participants started the study by completing the demographic questionnaire and the prior knowledge task. Afterward, learners received a pair of headphones and a music player and the learning phase began, during which the participants read or heard the text or the song about Henry VIII. To ensure sufficient learning outcome levels, the text was played twice in both auditory conditions. Lastly, students answered post-test questions, whilst half of them were listening to the melody of the sung version. At the end, the melody was scored by all students who listened to the melody during the post-test.

## Results

### Descriptive Data

Descriptive data for all variables in all conditions is shown in **Table 1**.

**Table 1:** Descriptive data for prior knowledge, recall, comprehension, all motivational scales, and the rating of the music per condition.

	Conditions												
	Text modality												
	Spoken				Sung				Written			
	Presentation of the melody during test phase												
	With (*n* = 19)		Without (*n* = 19)		With (*n* = 19)		Without (*n* = 19)		With (*n* = 19)		Without (*n* = 17)	
	*M*	*SD*	*M*	*SD*	*M*	*SD*	*M*	*SD*	*M*	*SD*	*M*	*SD*
Prior knowledge (%)	1.31	4.19	5.26	8.43	2.63	7.88	8.77	11.61	9.21	20.20	4.41	8.90
Recall (%)	45.96	13.03	48.25	20.23	49.47	15.29	51.40	18.44	62.28	19.57	66.27	16.49
Comprehension (%)	45.79	21.68	62.11	23.23	54.74	22.94	71.05	21.32	54.74	14.67	51.76	27.21
Current motivation^1^	20.11	3.07	20.36	2.91	19.52	2.53	20.57	2.79	19.68	2.58	19.27	2.05
Challenge^2^	5.03	1.15	5.05	1.61	5.02	1.01	5.01	0.95	5.04	1.13	4.63	1.25
Interest^2^	4.14	1.43	4.04	1.00	4.08	1.14	4.63	1.12	4.11	1.09	3.74	0.85
Probability of success^2^	5.58	0.88	5.60	0.91	5.20	1.01	5.46	1.22	5.35	1.12	5.59	1.00
Fear of failure^2^	2.64	1.28	2.33	1.22	2.86	1.64	2.54	1.45	2.82	1.39	2.68	2.64
The music was distracting^3^	3.44	1.41			2.95	1.45			2.68	1.45		
The music was stimulating^3^	1.88	0.89			2.39	1.07			2.58	1.22		
The music was pleasant^3^	3.19	1.11			3.00	1.37			3.52	1.26		

*^1^Range: 4–28, ^2^range: 1–7, ^3^range: 1–5.*

### Covariates

There were no significant differences between the learning conditions for any of the control variables (age, gender, prior knowledge, singing in a choir, playing an instrument, and liking classical music). However, as prior knowledge (with recall: *r* = 0.32, *p* < 0.01; with comprehension: *r* = 0.19, *p* < 0.05) and singing in a choir (with recall: *r* = 0.17, *p* < 0.05; with comprehension: *r* = 0.17, *p* < 0.05) were significantly correlated with learning outcomes they were considered as covariates in further analyses (see **Table 2**).

**Table 2:** Correlations between potential covariates and learning outcomes.

	Dependent variables			
	Recall		Comprehension	
	*r*	*p*	*r*	*p*
Age	–0.57	0.28	0.01	0.47
Gender	–0.56	0.28	–0.81	0.20
Prior knowledge	0.32	<0.001^***^	–0.19	0.03^*^
Singing in a choir	0.17	0.04^*^	0.17	0.04^*^
Playing an instrument	0.03	0.39	–0.01	0.39
Liking classical music	0.11	0.13	0.01	0.45

*^∗^*p* < 0.05; ^∗∗∗^*p* < 0.001.*

### Recall

**FIGURE 3:**
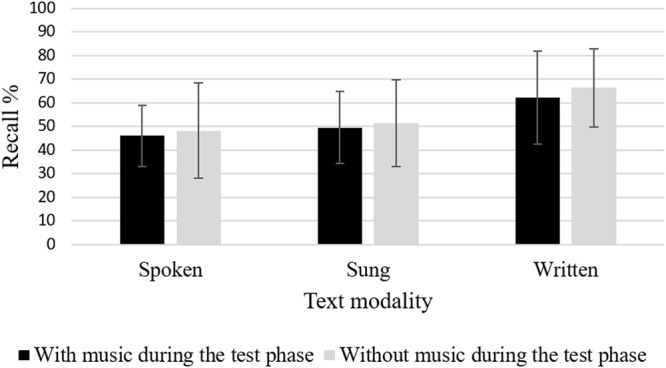
Recall performance (Error bars are standard deviations).

To test our hypotheses, we conducted an ANCOVA with the factors *text modality* and *presentation of the melody during the testing phase*. For recall, results of the ANCOVA showed significant effects for *text modality, F*(2,104) = 9.02, *p* < 0.001, $\eta_{p}^{2}$ = 0.15 (see **Figure 3**), but not for the *presentation of the melody during the test phase, F* < 1, ns. Planned *post hoc* contrasts showed that the visual text condition was superior to the sung text condition, *MD* = 0.14, *SE* = 0.04, *p* < 0.001, *d* = 0.78, as well as the spoken text condition, *MD* = 0.17, *SE* = 0.04, *p* < 0.001, *d* = 0.97. The spoken and the sung condition only differed on a descriptive level, *MD* = 0.04, *SE* = 0.04, ns. There was no significant interaction of the two independent variables, *F* < 1, ns.

### Comprehension

**FIGURE 4:**
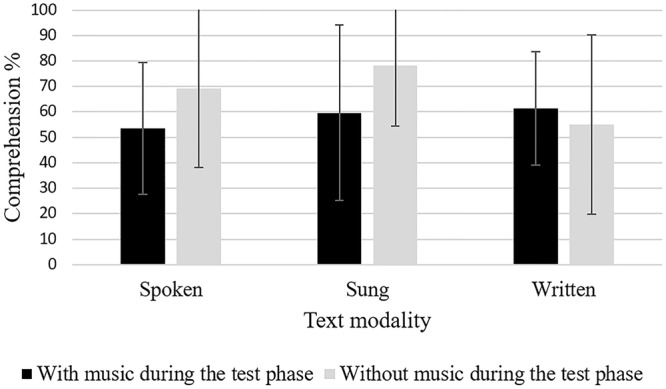
Comprehension performance (Error bars are standard deviations).

For comprehension, results of the ANCOVA showed significant effects for both *text modality, F*(2,104) = 3.16, *p* = 0.047, $\eta_{p}^{2}$ = 0.06 (see **Figure 4**), and the *presentation of the melody during the test phase, F*(1,104) = 4.15, *p* = 0.044, $\eta_{p}^{2}$ = 0.04 (see **Figure 4**). Listening to the melody during the test phase reduced comprehension performance. Planned *post hoc* contrasts showed a superiority of sung learning material over visual texts, *MD* = -0.11, *SE* = 0.05, *p* = 0.015, *d* = 0.40. The remaining two contrasts failed to show significant results: Sung versus auditive texts, *MD* = 0.07, *SE* = 0.05, ns, as well as auditive versus visual text, *MD* = -0.03, *SE* = 0.05, ns, only differed on a descriptive level. The interaction between the two independent variables were not significant, *F* < 1.

### Rating the Music

Participants, who listened to the melody during the test phase (*n* = 57), rated the melody as distracting to a medium level (*M* = 3.04, *SD* = 1.41), not very stimulating (*M* = 2.27, *SD* = 1.09), and rather pleasant (*M* = 3.24, *SD* = 1.27).

## Discussion

This study aimed to illustrate how three different types of learning material – spoken, sung, and visual text – differ concerning learning outcomes. Conforming our first hypothesis, the visual text led to the best test performance when recalling information. Reading gives the learner the possibility to self-regulate the learning process (e.g., Pächter, 1997). Learners can determine their reading speed themselves and are able to repeat single phrases if they need to. This seems to help while focusing on details which are necessary for high recall performance (Rubin et al., 2000). Moreover, reading a text was superior to listening to a text in a spoken or sung version for recall performance.

Even though these are significant findings, we did not find any advantage of the sung version over the spoken version. The dual coding of the sung text did not improve students’ performance. It may be that the modality of the learning material – visual or auditory – is the more important factor, which could outweigh the effect of the dual coded learning material. In this case, the disadvantage of listening to the text and as a consequence not focusing as much on details could not be offset by dual coding the sung version. At this point, it is important to note that the learning time for the spoken version was shorter than for the other versions, which could also have negatively influenced performance when recalling the spoken text (Cooper and Pantle, 1967; Kilgour et al., 2000).

Our second assumption pertaining to the first hypothesis stated that the sung version should lead to better comprehension outcomes than the other two versions. We did not expect differences between the visual and the spoken texts. Our results partly confirm this assumption. As predicted, the sung condition was significantly better than the visual text. Listening to a text, compared to reading a text lead to students gaining a better overview and more coherent understanding of the text, on which comprehension is based (Rubin et al., 2000). Moreover, comprehension processes are more complex than recall processes and, therefore, need more cognitive capacity. As cognitive capacity is limited (Miller, 1994; Sweller et al., 1998, 2011; Cowan, 2001), to increase comprehension levels it may be highly important to process the text in the modality which benefits comprehension. In addition, this result supports the idea that there is an additional subsystem for melodic information. Otherwise, the additional information of the melody in the sung text version, which also needs to be processed, would have led to higher cognitive burden and, therefore, to worse comprehension outcomes.

Contrary to the second assumption of our first hypothesis, sung and spoken texts differed only on a descriptive level, with better outcomes emerging after learning with the sung version. Even though the spoken text does not have the advantage of being dually coded, the difference was not statistically significant. Both spoken and sung learning materials are auditory and, therefore, foster comprehension. It seems as though this common advantage dominates out any other differences between both modalities.

Another important factor in this investigation is the motivation induced by the sung text. Depending on how stimulating and pleasant a melody is, a learner’s motivation may increase or decrease, thus affecting learning outcomes (Vollmeyer and Rheinberg, 2006). In this study, our students rather liked the melody and scored it as being fairly stimulating. In addition, the situation of learning with a song was absolutely new and a welcome change to the everyday school routine. Thus could have fostered motivation and, therefore, increased learning outcomes as well. It might be quite counterintuitive to think that listening to songs should foster deeper understanding and comprehension of its lyrics. There is an everyday phenomenon of listening to or even singing a song, without being aware of the semantic meaning of the song’s lyrics. The differences to our study might be explained by two different factors. First, for all non-native English-speaking countries, most of the time songs are not in the listeners’ mother tongue. Second, the instructions in our study matter. Listening to a song in everyday life is usually not the main, but a secondary task. Therefore, usually a listener’s attention is not entirely focused on understanding the lyrics. Our students were explicitly informed that they would have to answer questions about the lyrics after the learning phase. Therefore, the motivation to semantically process the lyrics and the goal (learning) was a completely different one than traditionally faced with when listening to music in everyday life.

To conclude the first research question, this is the first study to compare these three modalities in terms of different levels of learning outcomes. Earlier studies either focused on visual versus auditive, or auditive versus sung texts, with only recall performance being measured. Furthermore, until this point no study had included both visual and sung texts. In addition, our participants were students, whilst earlier studies collected data from the adult population. Thus, practical implications emerging from this study are relevant for high school education. These will be discussed below.

With our second research question we investigated mnemonic or disturbing effects of music while retrieving information during the test phase. Our second hypothesis stated that we would not find a main effect of the presentation of the melody during the test phase. We postulated that there would, instead, be an interaction with the text modality while learning, as stated in hypothesis 3. Confirming our hypotheses, listening to the melody did not influence recall performance in general, but no interaction between the two factors was apparent. We did not expect to find a main effect, as the group that learned with the song should profit from listening to the melody, while both other groups’ learning performance should become worse. This explanation must be dismissed, however, as the interaction between both factors is not significant. One might argue that answering recall questions does not require too much cognitive capacity so that the distracting effect of a piece of unknown music is not too strong. Nevertheless, the melody did not function as an anchor to improve recall. One possible explanation to this is that the melody may not have been specific enough to activate the line of the lyrics which needed to be recalled. Each verse was sung to the same sound sequence. Thus, hearing one sequence simultaneously activated each parallel line of every verse and therefore could have led to interferences. One can compare this phenomenon to common mnemonics, such as the loci method (Bower, 1970). If one imagines several different objects and links them all to the same place, thinking of this place would not benefit recall of a specific object. In contrast, the memory of all these objects would be activated and the parallel activated memory traces would interfere and hinder retrieval. Summing up, listening to the melody during the test phase did not influence recall performance, independent of the experimental group.

For comprehension outcomes, neither hypotheses (H2 and H3a/b) were validated. Participants who learned with either the auditive or musical learning material showed worse learning outcomes when they listened to the melody during the test phase than the group that did not listen to the melody. Within the visual modality, neither group differed statistically in terms of comprehension. This result could be expected for the auditive group, as the music did not work as a mnemonic anchor and hence, must have led to an additional load of processing. This leads to cognitive overload worsening learning outcomes. But how does this correspond to the notion of an independent subsystem for the processing of melodies (Deutsch, 1970; Rowe et al., 1974; Salame and Baddeley, 1989; Berz, 1995)? One possible explanation is that this subsystem is not completely independent but integrated in the phonological loop. Thus, listening to and processing the melody would have a reduced, but still noticeable influence on the main task. This is especially the case as the phenomenological loop is strongly involved in answering questions which are formulated in words. This may seem contradictory to the fact, that learning with the sung text did foster comprehension because the melody needs to be processed in the phonological loop as well. In this case, the melody and the text were highly linked as they formed a song. As a result, they were both constructed on the same melodic pattern. Therefore, both pieces of information are paired and can be processed not only simultaneously in parallel but also together. Contrary to our assumptions, in the group which learned with the sung text, listening to the melody during testing also led to worse test performance. As already discussed in the recall section, the melody was not linked to the lyrics particularly successfully in this experiment. Thus, listening to the melody could not have a positive influence on comprehension performance, rather the same negative influence as in all other groups. When listening to the melody during the test phase the visual group showed slightly better results on a descriptive level. This might be due to motivational effects; during the learning phase this group did not hear any interesting information through the headphones, only the instructions to start and stop reading. Hence, finally listening to the interesting melody might have increased their engagement. Furthermore, de Groot (2006) postulates that individuals differ concerning their ability to benefit from background music: While some people’s performance can potentially be raised by background music, others show a decrease in performance (see also Lehmann and Seufert, 2017).

## Conclusion

We cannot say whether it is possible to use the melody of a song as a mnemonic because of the characteristics of the melody used in this experiment. In future studies, melodies should be used which differ between every single line so as to be specific enough to function as an anchor. The ability to truly learn all of the different lines would probably only work for short texts. Furthermore, it is important to confirm that the melody is easy enough to be learned within the time constraints of the experiment, and the song should be repeated a sufficient amount of times, so the learner can use it successfully as a mnemonic.

Building on the fact that the melody did not function as an anchor, the question arises as to why the music during the test phase did not have a general negative influence on recall but only on comprehension performance. Based on Bloom’s (1956) taxonomy, answering recall questions is easier than answering comprehension questions and thus, needs less cognitive capacity. Therefore, there is enough cognitive capacity left to process the melody, whilst simultaneously answering recall questions. Only when answering the more challenging comprehension questions does the negative impact of listening to the melody carry weight.

### Practical Implications

As a practical implication, we definitely do not recommend listening to a melody while concentrating on answering a learning task. Even if the negative influence only impacts more challenging tasks, we were not able to show any positive influences of listening to a melody on any level. Nevertheless, learner’s characteristics may play an important role in this link. Further research should, therefore, focus on particular requirements a learner may need to fulfill to be successful when learning with music, such as personality or working memory capacity.

For practical advice in which modality a text should be presented in, our results can be considered to be more controversial. On the one side, songs for academic learning are worthwhile for fostering comprehension, however, on the other side, songs for learning are time-consuming to design. Each melody needs to be composed and the lyrics written to fit the music, which is much more effort than simply writing a text. This is made more pertinent when we see that that the learning outcomes emerging from the spoken and the sung versions only differed on a descriptive level. In addition, one must not forget that recall performance become worse when learning with songs, so they cannot be used independently of the learning target. Considering the effort involved in producing a song for learning, they should only be used for specific occasions, such as with very important topics which are difficult to understand. Bearing in mind the effortful production of a song, we would recommend sharing produced songs as open educational resources between teachers and schools, so that anyone could receive access. Educational films and animations, for example, are also time consuming to produce. This is why teachers mostly do not produce them on their own, but rely on the pool of available material.

### Limitations and Further Research

In terms of limitations, one critical point is that the prior knowledge of our participants was very limited and with little variation. Learning outcomes may be different for groups with a higher level of prior knowledge or for groups which varies more. Additionally, the role of motivation is vague. Motivation could function as a mediator variable and should be measured in upcoming studies. In this study, participants were really curious about taking part in our experiment and reported that they had welcomed this diversion from everyday school routine. As learning with songs was particularly new to them, this group might have benefited the most from this motivational impact. However, using songs on a regular basis could cancel out this effect.

Furthermore, the format of the visual text was quite unusual because it was rhymed. As song lyrics are usually rhymed and because we wanted to keep with this format, it was necessary to present the visual text with the very same words. Only in this way, is it possible to compare results without any other effects induced by differences in the wording.

Moreover, the role of cognitive load should be investigated further as three questions have emerged from our study. Firstly, how do the three types of learning material differ in terms of any extraneous and germane cognitive load which may arise during learning? Secondly, how does listening to the melody during the test phase impacts extraneous cognitive load? Lastly, does a learner’s preference for either visual or auditory learning material influence learning outcomes in this context?

Another interesting point is the level of familiarity with the melody. In this study, participants only listened to the melody twice. Thus, they had no chance to learn the complete sequence of tones. Different results may be found when lyrics are presented to a well-known melody that can be recalled freely by the learners. Such a melody, that is completely stored in long-term memory, may work better as a mnemonic aid and thereby foster recall performance of the lyrics.

In addition, the role of musical training in learning with music is an interesting avenue for further study. We found a positive correlation between singing in a choir and recall and comprehension for all groups. One reason for this might be the fact that participants with higher levels of musical expertise may have found it easier to learn the melody of the song. Therefore, it might have been easier for them to use it as a mnemonic aid. Moreover, it is possible that ongoing musical education could not only foster learning with songs, but also learning in general. Similar results were already found for recall outcomes in a study by Kilgour et al. (2000). This should be further investigated in studies using a more differentiated measure of musical experience.

## Author Contributions

JL designed and conducted this study and wrote this manuscript – all under supervision of TS.

## Conflict of Interest Statement

The authors declare that the research was conducted in the absence of any commercial or financial relationships that could be construed as a potential conflict of interest.
